# Effects of aromatic compounds on the production of bacterial nanocellulose by *Gluconacetobacter xylinus*

**DOI:** 10.1186/1475-2859-13-62

**Published:** 2014-04-30

**Authors:** Shuo Zhang, Sandra Winestrand, Xiang Guo, Lin Chen, Feng Hong, Leif J Jönsson

**Affiliations:** 1 China-Sweden Associated Research Laboratory in Industrial Biotechnology, College of Chemistry, Chemical Engineering and Biotechnology, Donghua University, Shanghai 201620, China; 2 Group of Microbiological Engineering and Industrial Biotechnology, College of Chemistry, Chemical Engineering and Biotechnology, Donghua University, Shanghai 201620, China; 3 Department of Chemistry, Umeå University, Umeå SE-901 87, Sweden; 4 School of Environmental Science & Engineering, Donghua University, Shanghai 201620, China

**Keywords:** *Gluconacetobacter xylinus*, Phenolic compound, Bacterial cellulose, Inhibitor

## Abstract

**Background:**

Bacterial cellulose (BC) is a polymeric nanostructured fibrillar network produced by certain microorganisms, principally *Gluconacetobacter xylinus.* BC has a great potential of application in many fields. Lignocellulosic biomass has been investigated as a cost-effective feedstock for BC production through pretreatment and hydrolysis. It is well known that detoxification of lignocellulosic hydrolysates may be required to achieve efficient production of BC. Recent results suggest that phenolic compounds contribute to the inhibition of *G. xylinus*. However, very little is known about the effect on *G. xylinus* of specific lignocellulose-derived inhibitors. In this study, the inhibitory effects of four phenolic model compounds (coniferyl aldehyde, ferulic acid, vanillin and 4-hydroxybenzoic acid) on the growth of *G. xylinus,* the pH of the culture medium, and the production of BC were investigated in detail. The stability of the phenolics in the bacterial cultures was investigated and the main bioconversion products were identified and quantified.

**Results:**

Coniferyl aldehyde was the most potent inhibitor, followed by vanillin, ferulic acid, and 4-hydroxybenzoic acid. There was no BC produced even with coniferyl aldehyde concentrations as low as 2 mM. Vanillin displayed a negative effect on the bacteria and when the vanillin concentration was raised to 2.5 mM the volumetric yield of BC decreased to ~40% of that obtained in control medium without inhibitors. The phenolic acids, ferulic acid and 4-hydroxybenzoic acid, showed almost no toxic effects when less than 2.5 mM. The bacterial cultures oxidized coniferyl aldehyde to ferulic acid with a yield of up to 81%. Vanillin was reduced to vanillyl alcohol with a yield of up to 80%.

**Conclusions:**

This is the first investigation of the effect of specific phenolics on the production of BC by *G. xylinus*, and is also the first demonstration of the ability of *G. xylinus* to convert phenolic compounds. This study gives a better understanding of how phenolic compounds and *G. xylinus* cultures are affected by each other. Investigations in this area are useful for elucidating the mechanism behind inhibition of *G. xylinus* in lignocellulosic hydrolysates and for understanding how production of BC using lignocellulosic feedstocks can be performed in an efficient way.

## Background

In recent years bacterial cellulose (BC), a cellulosic material obtained through a microbial process, has received increasing attention. Unlike the cellulose of plants, BC has an ultrafine nanofiber network. It is synthesized by some species of bacteria, especially *Gluconacetobacter xylinus* (formerly *Acetobacter xylinus*). *G. xylinus* is a Gram-negative, obligately aerobic rod-shaped bacterium*,* with good capability to produce BC [1]. BC has unusual and characteristic physicochemical and mechanical properties, such as high purity (free of lignin and hemicelluloses), high degree of polymerization, large surface area, excellent tensile strength, high porosity, and good biocompatibility. Due to its unique features, BC has been found to be useful in many diverse fields including textile, food and waste treatment [2], but especially in the field of biomedical materials, which include artificial blood vessels [3] or vascular graft materials [4,5], temporary wound dressing [6], and bone grafting [7].

In order to decrease the production cost of BC, attempts have been made to find cost-effective carbon feedstocks for BC production. That would facilitate utilization of BC outside the medical area, in which the cost of the BC is less important. In recent years, renewable biomass, such as lignocellulosic resources, has been most studied as potential feedstock. Biomass resources that have been investigated include konjak glucomannan [8], rice bark [9], wheat straw [10-12], cotton-based waste textiles [13,14], waste fiber sludge [15] and spruce [16]. The biomass is typically hydrolyzed enzymatically, since this approach gives high sugar yields. Before enzymatic hydrolysis, lignocellulosic biomass is pretreated to make the cellulose more accessible to cellulolytic enzymes. A typical pretreatment will result in the formation of by-products such as aliphatic acids, furan aldehydes, and phenolic compounds [17]. In sufficiently high concentrations, these by-products will inhibit microorganisms, bacteria as well as yeasts. While relatively high concentrations of aliphatic acids and furan aldehydes are required to negatively influence yeast, some phenolic compounds are strongly inhibitory even at low concentrations [17,18]. With regard to *G. xylinus*, it is well known that detoxification of lignocellulosic hydrolysates may be required to achieve efficient production of BC [10]. Recent results suggest that phenolic compounds contribute to the inhibition of *G. xylinus*[16]. However, very little is known about the effect on *G. xylinus* of specific lignocellulose-derived inhibitors. This study addresses that lack of knowledge, and is focused on the effect of phenolic compounds derived from lignocellulosic biomass.

**Figure 1:**
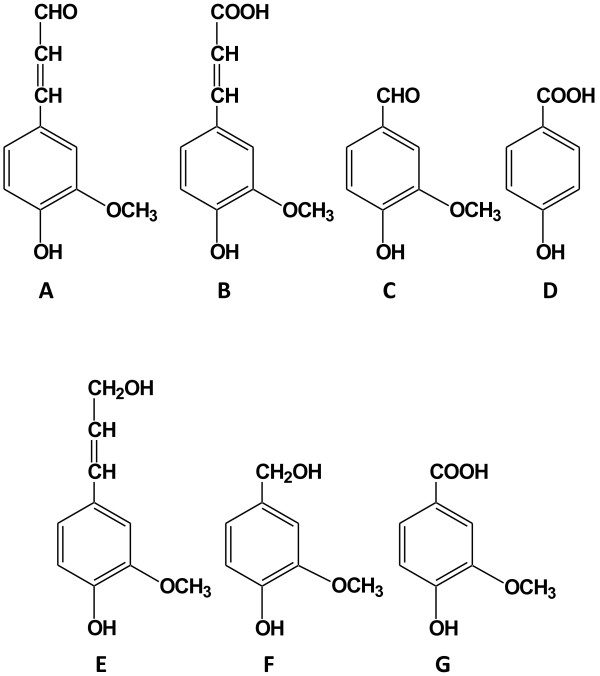
**The structure of model inhibitors and related compounds. (A)** coniferyl aldehyde, **(B)** ferulic acid, **(C)** vanillin, **(D)** 4-hydroxybenzoic acid, **(E)** coniferyl alcohol, **(F)** vanillyl alcohol, and **(G)** vanillic acid.

The influence of four phenolic model inhibitors was investigated with regard to the growth of *G. xylinus*, the sugar consumption, the change of pH during cultivation, the cell viability, and the yield of BC. The experimental approach applied some modern analytical techniques including high-performance liquid chromatography equipped with a UV detector and a diode array and multiple wavelength detector (HPLC-UV-DAD) for analysis of phenols, fluorescence staining for analysis of cell viability, and enzyme technology for analysis of sugar consumption. Furthermore, potential biotransformation of the inhibitory phenolics during cultivation was also studied. The four phenolic model compounds (Figure 1A-D) included two aldehydes, coniferyl aldehyde and vanillin, and two carboxylic acids, ferulic acid and 4-hydroxybenzoic acid. Coniferyl aldehyde has been identified in spruce hydrolysates and has been used extensively as a model compound to study the effect of inhibition of production of cellulosic ethanol by the yeast *Saccharomyces cerevisiae*[19-21]. Vanillin is one of the most prevalent phenolic compounds in lignocellulosic hydrolysates and has been identified in for example hydrolysates from spruce [19,20], pine, poplar, corn stover [22], wheat straw [23], and sugarcane bagasse [24]. Ferulic acid and 4-hydroxybenzoic acid are common in various hydrolysates, for example from spruce, pine, poplar, corn stover and sugarcane bagasse [20,22,24]. This is the first study of the effect of specific phenolics on the production of BC by *G. xylinus*. Investigations in this area are useful for elucidating the mechanism behind inhibition of *G. xylinus* by lignocellulosic hydrolysates and for understanding how production of BC using lignocellulosic feedstocks can be performed in an efficient way.

## Results

**Figure 2:**
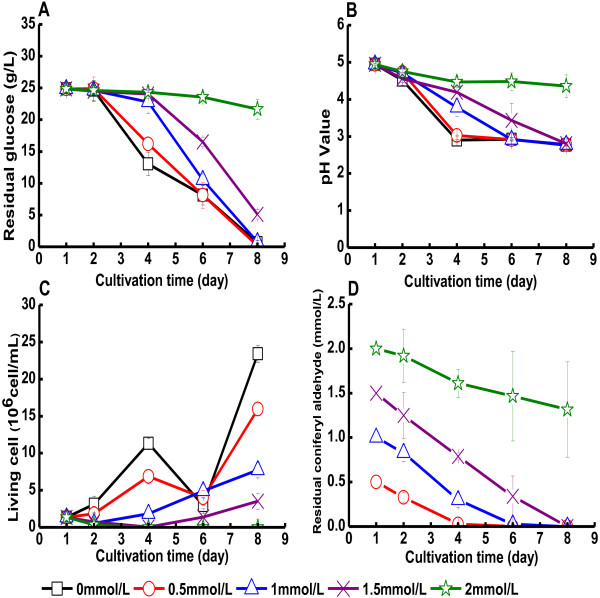
**Cultivation of *G. xylinus *in medium containing coniferyl aldehyde.** The figure shows changes in **(A)** the glucose concentration in the culture medium, **(B)** the pH value of the culture medium, **(C)** the concentration of living cells, and **(D)** the concentration of coniferyl aldehyde. Coniferyl aldehyde was added on day one. Error bars show standard errors of means of three replicates.

Results from cultivations of *G. xylinus* in the presence of coniferyl aldehyde are shown in Figure 2 and Table 1. The glucose consumption rates in cultures with initial concentrations of coniferyl aldehyde of 0.5 mM, 1.0 mM and 1.5 mM were 3.5 g/[L · d], 3.4 g/[L · d] and 2.8 g/[L · d], respectively. This was relatively close to the glucose consumption rate of the culture with reference medium, which was 3.5 g/[L · d] (Table 1A), although a slight inhibition was observed at concentrations of 1.0 and 1.5 mM coniferyl aldehyde. At 2.0 mM coniferyl aldehyde, the glucose consumption rate dropped drastically to 0.45 g/[L · d]. The concentration of live bacteria decreased as the concentration of coniferyl aldehyde increased (Figure 2C). At the end of the cultivation, the pH decreased to 2.8, which was the same as for the reference medium, except for cultures with 2.0 mM coniferyl aldehyde for which there was not much change in pH (Figure 2B). For cultures with 0.5-1.5 mM coniferyl aldehyde, the volumetric yield of BC was in the range 3.4-6.4 g/L, which was lower than that of the culture with reference medium (6.7 g/L) (Table 1B). No BC production was detected in cultures with 2.0 mM coniferyl aldehyde. The yield of BC on consumed glucose showed the same trend. Increasing coniferyl aldehyde concentrations from 0.5 to 1.5 mM resulted in a decrease of the yield of BC from 0.26 to 0.17 g/g, while the reference medium gave a BC yield of 0.28 g/g (Table 1C). At the end of the cultivation, all coniferyl aldehyde was converted except for cultures with an initial concentration of coniferyl aldehyde of 2 mM where most of it remained (Figure 2D).

**Table 1:** **Glucose consumption rates and yields of bacterial cellulose in *Gluconacetobacter xylinus *** **cultures after 7 d**^**a**^

**A. Glucose consumption rates (g/[L∙d])**									
**Phenols**	**Concentration (mM)**								
	**0**	**0.5**	**1.0**	**1.5**	**2.0**	**2.5**	**5**	**7.5**	**10**
Coniferyl aldehyde	3.5±0.1	3.5±0.1	3.4±0.1	2.8±0.1	0.45±0.01	-	-	-	-
Ferulic acid	3.5±0.1	3.5±0.1	3.5±0.1	3.5±0.3	3.5±0.1	-	-	-	-
Vanillin	3.5±0.1	3.3±0.1	-	-	-	1.7±0.9	0.31±0.13	0.37±0.05	0.34±0.09
4-Hydroxybenzoic acid	3.5±0.1	3.4±0.1	-	-	-	3.5±0.1	3.5±0.1	3.4±0.1	3.3±0.1
**B. Volumetric yield of bacterial cellulose (g/L)**									
**Phenols**	**Concentration (mM)**								
	**0**	**0.5**	**1.0**	**1.5**	**2.0**	**2.5**	**5**	**7.5**	**10**
Coniferyl aldehyde	6.7±0.3	6.4±1.0	4.2±1.4	3.4±1.0	ND^b^	-^c^	-	-	-
Ferulic acid	6.7±0.3	6.4±0.5	6.5±0.2	6.1±0.5	6.6±2.1	-	-	-	-
Vanillin	6.7±0.3	5.8±0.3	-	-	-	2.9±0.3	0.38±0.27	0.35±0.40	0.07±0.08
4-Hydroxybenzoic acid	6.7±0.3	6.4±1.1	-	-	-	6.2±0.6	5.9±0.5	5.4±1.6	5.0±0.4
**C. Bacterial cellulose yield on consumed glucose (g/g)**									
**Phenols**	**Concentration (mM)**								
	**0**	**0.5**	**1.0**	**1.5**	**2.0**	**2.5**	**5**	**7.5**	**10**
Coniferyl aldehyde	0.28±0.01	0.26±0.04	0.17±0.06	0.17±0.05	ND	-	-	-	-
Ferulic acid	0.28±0.01	0.26±0.02	0.26±0.01	0.25±0.02	0.27±0.08	-	-	-	-
Vanillin	0.28±0.01	0.25±0.01	-	-	-	0.24±0.05	0.17±0.06	0.13±0.08	0.03±0.01
4-Hydroxybenzoic acid	0.28±0.01	0.29±0.05	-	-	-	0.25±0.02	0.24±0.02	0.22±0.06	0.21±0.01

^a^Three parallel cultures were studied for each inhibitor concentration. The table shows mean values ± standard errors.

^b^None detected.

^c^Not studied.

Control experiments (without bacterial inoculation but with addition of coniferyl aldehyde) showed no conversion products from coniferyl aldehyde. Analysis of the culture medium indicated that a large part of the coniferyl aldehyde was transformed to ferulic acid (Table 2). The highest yield of ferulic acid was 81% and was detected in cultures with an initial coniferyl aldehyde concentration of 1.5 mM. Small amounts of coniferyl alcohol (Figure 1E) were also detected in some of the cultures, but in samples taken at the end of the cultivation coniferyl alcohol was only detected in cultures with initial coniferyl aldehyde concentrations of 1.5 and 2.0 mM, and the coniferyl alcohol concentrations were <0.1 mM. Thus, coniferyl alcohol was probably present as an intermediate bioconversion product.

**Table 2:** **Conversion of coniferyl aldehyde and vanillin in *Gluconacetobacter xylinus *** **cultures**^**a**^

**Initial concentration of coniferyl aldehyde (mM)**	**0.50**	**1.0**	**1.5**	**2.0**	
Yield of ferulic acid (%)	74	74	81	44	
**Initial concentration of vanillin (mM)**	**0.50**	**2.5**	**5.0**	**7.5**	**10**
Yield of vanillyl alcohol (%)	80	53	ND^b^	ND	ND

^a^The table shows the yields of ferulic acid at the end of cultivation of cultures with coniferyl aldehyde, and the yields of vanillyl alcohol at the end of cultivation of cultures with vanillin. Yield of ferulic acid (%) = ferulic acid / original coniferyl aldehyde, Yield of vanillyl alcohol (%) = vanillyl alcohol / original vanillin.

^b^None detected.

**Figure 3:**
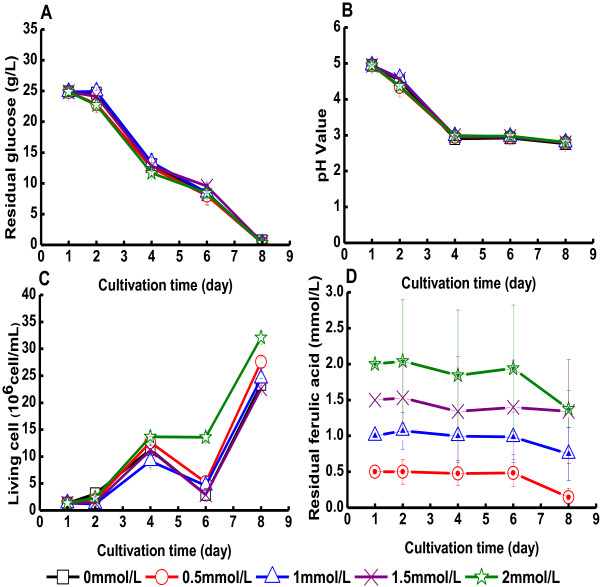
**Cultivation of *G. xylinus *in medium containing ferulic acid.** The figure shows changes in **(A)** the glucose concentration in the culture medium, **(B)** the pH value of the culture medium, **(C)** the concentration of living cells, and **(D)** the concentration of ferulic acid. Ferulic acid was added on day one. Error bars show standard errors of means of three replicates.

The effects of addition of ferulic acid to bacterial cultures are shown in Figure 3 and Table 1. Figure 3 indicates that addition of ferulic acid to the medium to concentrations of up to 2 mM did not negatively affect the cultures of *G. xylinus*. Furthermore, Table 1 indicates that neither glucose consumption rate nor BC yield were affected to any larger extent. The concentration of living cells was higher in the presence of 2 mM ferulic acid, which suggests that it could slightly enhance bacterial growth (Figure 3C). At the end of the cultivation, the concentration of living bacteria in medium with 2 mM ferulic acid was 32.1 × 10^6^ cells/mL, while the reference medium without ferulic acid only contained 23.4 × 10^6^ cells/mL. This was not due to a buffering effect, as the pH decreased to 2.8 in all the media (Figure 3B). The concentration of ferulic acid did not change very much during the cultivation (Figure 3D). Most cultures exhibited a decline in the concentration of ferulic acid at the end of the cultivation, but it is not clear that it was significant.

**Figure 4:**
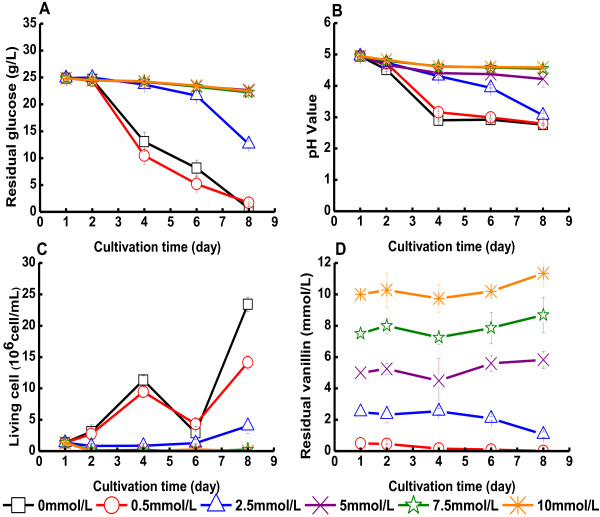
**Cultivation of *G. xylinus *in medium containing vanillin.** The figure shows changes in **(A)** the glucose concentration of the culture medium, **(B)** the pH value of the culture medium, **(C)** the concentration of living cells, and **(D)** the concentration of vanillin. Vanillin was added on day one. Error bars show standard errors of means of three replicates.

Results obtained with vanillin are shown in Figure 4 and in Table 1. Figure 4 indicates that vanillin did not severely inhibit the growth of *G. xylinus* until the concentration exceeded 2.5 mM (Figure 4A-C). An initial vanillin concentration of 0.5 mM hardly affected glucose consumption, pH value or BC yield (Figure 4 and Table 1). With an initial vanillin concentration of 2.5 mM, the glucose consumption rate declined from 3.5 g/(L∙d) to 1.7 g/(L∙d) (Table 1A). The concentration of living bacteria at the end of the cultivation was 4.0 × 10^6^ cells/mL, much lower than in the cultures with reference medium, which reached 23.4 × 10^6^ cells/mL (Figure 4C). The volumetric yield of BC declined as the concentration of vanillin increased (Table 1B). When the initial concentration of vanillin was 5 mM or higher, the glucose consumption rate was <0.4 g/(L∙d) (Table 1A), the concentration of living bacteria in the cultures was very low (Figure 4C), and the yield of BC was <0.4 g/L (Table 1B).

The vanillin content in cultures with initial concentrations of 0.5 or 2.5 mM seemed to decrease during the cultivation (Figure 4D). Control experiments (with vanillin in the medium but without bacterial inoculation) showed no product formation making it plausible that conversion products detected in bacterial cultures would be the result of a biotransformation. Only very small concentrations of vanillic acid (Figure 1G) were detected, and the main product was instead vanillyl alcohol (Figure 1F). The highest yield of vanillyl alcohol at the end of the cultivation was 80% and was found in cultures with an initial vanillin concentration of 0.5 mM (Table 2). Most of the vanillin had been reduced to vanillyl alcohol also in cultures with an initial vanillin concentration of 2.5 mM, but at higher vanillin concentrations no vanillyl alcohol was detected.

**Figure 5:**
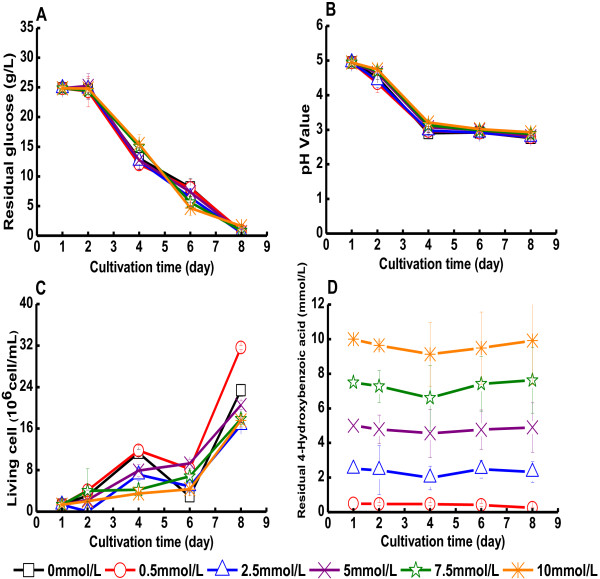
**Cultivation of *G. xylinus *in medium containing 4-hydroxybenzoic acid.** The figure shows changes in **(A)** the glucose concentration of the culture medium, **(B)** the pH value of the culture medium, **(C)** the concentration of living cells, and **(D)** the concentration of 4-hydroxybenzoic acid. 4-Hydroxybenzoic acid was added on day one. Error bars show standard errors of means of three replicates.

The results of experiments with 4-hydroxybenzoic acid are shown in Figure 5 and in Table 1. In the concentration range studied, 0.5-10 mM, 4-hydroxybenzoic acid did not show any clear negative effect on the glucose consumption, the culture pH, or the cell viability of *G. xylinus* (Figure 5A-C). The concentration of 4-hydroxybenzoic acid did not change significantly during the cultivation (Figure 5D). The yield of BC slightly decreased as the concentration of 4-hydroxybenzoic acid increased (Table 1). The lowest yield of BC, 5.0 g/L, was obtained in medium with 10 mM 4-hydroxybenzoic acid (Table 1B). The BC yield on consumed glucose also declined with increasing concentrations of 4-hydroxybenzoic acid, which means that the cells to an increasing degree utilized glucose for other purposes than production of BC when the concentration of the acid increased.

## Discussion

A comparison of the effects of four phenolic compounds commonly found in lignocellulosic hydrolysates showed that coniferyl aldehyde was most toxic for *G. xylinus.* The main bioconversion product of coniferyl aldehyde was ferulic acid, which is formed by oxidation of the aldehyde group to a carboxylic acid group. As ferulic acid was found to be less toxic than coniferyl aldehyde, this biotransformation will serve as a detoxification reaction. In a study with the bacterium *Pseudomonas* sp strain HR 199, coniferyl aldehyde dehydrogenase was found to be responsible for intracellular conversion of coniferyl aldehyde to ferulic acid [25]. The bacterium *Acetobacter aceti* MIM2000/28 has also been reported to have the ability to oxidize aldehydes into corresponding carboxylic acids [26]. *Acetobacter* and *Gluconobacter* are two genera of acetic acid bacteria [27] and can be expected to have similarities with regard to their metabolism. While ferulic acid was clearly the predominant product, small amounts of coniferyl alcohol were also detected in cultures of *G. xylinus* (this work). The results indicate that biotransformation by an oxidation step is preferred, but that a reduction may also take place. Oxidation and reduction of aromatic aldehydes in parallel has also been observed for the ascomycete fungus *Aureobasidium pullulans*[28]*.* The bacterium *Gluconobacter oxydans* is known to possess several membrane-bound dehydrogenases including an aldehyde dehydrogenase [29]. Therefore it can be speculated that *G. xylinus* may also have this kind of oxidoreductases and a hypothetical reaction scheme would be:

$$\begin{array}{ll} \mathrm{Coniferyl}\;\mathrm{aldehyde} & +H_{2}O+\mathrm{NAD}{\left(P\right)}^{+}\to {\left[\mathrm{Ferulate}\right]}^{‒}\\ & +\mathrm{NAD}\left(P\right)H+2H^{+} \end{array}$$

Ferulic acid exhibits structural similarities with coniferyl aldehyde (Figure 1), but did not inhibit the growth of the bacterium or the production of BC. Instead, cell viability was slightly stimulated at a concentration of ferulic acid of 2 mM. The smaller carboxylic acid studied, 4-hydroxybenzoic acid, was less toxic than vanillin, which is similar except that it has an aldehyde group instead of a carboxylic acid group and also a methoxyl group (Figure 1). Thus, the aromatic acids included in this study were less toxic than similar aldehydes. It is possible that an acetic acid bacterium as *G. xylinus* can tolerate phenolic acids due to that it has a well developed capability to resist high concentration of carboxylic acids. Previous studies have also indicated that phenolic aldehydes are more toxic than the corresponding carboxylic acids and aromatic alcohols for *S. cerevisiae*[18], *Candida utilis*, *C. albicans*[30] and *Klebsiella pneumoniae*[31,32]. The pKa values of ferulic acid and 4-hydroxybenzoic acid are 4.58 and 4.54, respectively. That means that they were predominately deprotonated in the beginning of the experiments (at pH 5.0), but that they were protonated in the end of the experiments when the pH of the bacterial cultures had dropped to about 3. It would be reasonable to assume that the protonated form is more toxic, as it is less hydrophilic than the deprotonated form and may more easily pass through the cell membrane. Therefore, the concentrations of the toxic form of the carboxylic acids may initially have been lower than in comparable experiments with the aldehydes, which agrees with the observation that the effect on the cultures was less obvious for the acids.

*G. xylinus* was rather sensitive to vanillin, since growth and BC production were almost completely inhibited already at a concentration of 5 mM. This can be compared with the *S. cerevisiae* strain BY4743, the growth of which was inhibited by approx. 50% when the concentration of vanillin was 5 mM [33]. Coniferyl aldehyde was more inhibitory than vanillin though both of them have only one aldehyde group. However, coniferyl aldehyde has an unsaturated bond in its propanoid backbone (Figure 1A). This structure has been reported to be a major contributor to the inhibitory effect of phenolic compounds on *S. cerevisiae*[18,34], and in accordance with our results this observation appears valid also for *G. xylinus*.

The concentrations of vanillic acid in the *G. xylinus* cultures were very low (<0.1 mM). Vanillin and coniferyl aldehyde are structurally related (Figure 1), but surprisingly the reduction product vanillyl alcohol was predominant in *G. xylinus* cultures with vanillin rather than the oxidation product vanillic acid. Vanillin has been found to be reduced to vanillyl alcohol by yeast [18]. *Klebsiella pneumoniae* also reduces vanillin to vanillyl alcohol [32]. However, *Pseudomonas fluorescens* strain BTP 9 was found to convert vanillin into vanillic acid [35]. *Gluconobacter oxydans* subsp. *suboxydans* ATCC 621 was reported to have the ability to convert low concentrations of vanillin, but with higher concentrations it needed prolonged cultivation time to overcome the inhibition [29]. The biotransformation products of vanillin by *G. oxydans* cultures were found to be vanillic acid and vanillyl alcohol. The ratio of vanillic acid to vanillyl alcohol was around 3:1, and the enzymes responsible for the transformations may be induced by vanillin in the medium [29]. It is likely that different cultivation conditions can contribute to the different product formation patterns observed with various microbial strains, but an interesting point with respect to our study is that although the microbial strain was the same and the cultivation conditions for the experiments with coniferyl aldehyde and vanillin were the same the product formation patterns were opposite with regard to oxidative and reductive biotransformations. This suggests that the catalytic efficiency of the *G. xylinus* ATCC 23770 enzymes partaking in the oxidative biotransformations is higher for coniferyl aldehyde than for vanillin, while the opposite would be expected for reductive biotransformation.

Kubiak *et al.*[36] investigated the complete genome sequence of *G. xylinus* E25 and identified a megaplasmid that had not been reported before. Megaplasmids have been found to be important for the survival of other *Alphaproteobacteria* genera in unfavorable environments. Kubiak *et al.*[36] also found some *G. xylinus* E25 genes connected to oxidoreductases, for instance H845_1089 and H845_1144. The activity of enzymes coded for by such genes may help to explain the ability of *G. xylinus* to grow in medium containing aromatic compounds. The potential effects of dehydrogenases and oxidoreductases on aromatic compounds would be of interest for future studies.

## Conclusions

Of four lignocellulose-derived phenolics studied, coniferyl aldehyde was more inhibitory than vanillin, ferulic acid, and 4-hydroxybenzoic acid. The phenolic carboxylic acids included in the study tended to be less inhibitory to *G. xylinus* than the phenolic aldehydes, but in part that may be an effect of the initial pH of the microbial cultures. To get a better view of the inhibitory effects of the acids, cultivation experiments performed using controlled pH would be of interest to perform in the future. It is however clear that phenolic aldehydes are potent inhibitors of *G. xylinus*, and therefore it should be important to minimize their formation during pretreatment or to use detoxification methods that target this type of compounds. In the long run, the bacterium may be helped by its ability to convert phenolic aldehydes to less toxic compounds. An interesting observation is that the main biotransformation products of *G. xylinus* cultures with coniferyl aldehyde and vanillin were ferulic acid and vanillyl alcohol, respectively. It would be of interest to perform further studies of the enzymes that are responsible for oxidative and reductive biotransformation of phenolic aldehydes in *G. xylinus*. The effects of other types of inhibitors on *G. xylinus*, for example aliphatic acids and furan aldehydes, and potential synergistic effects of different inhibitors also deserve further attention.

## Methods

### Materials and microorganism

Coniferyl aldehyde (≥98%), ferulic acid (≥99%), vanillin (≥99%), 4-hydroxybenzoic acid (≥99%), coniferyl alcohol (≥98%), vanillyl alcohol (≥98%), and vanillic acid (≥97%) (Figure 1) were purchased from Sigma-Aldrich (Steinheim, Germany). *Gluconacetobacter xylinus* ATCC 23770 was obtained from the American Type Culture Collection (Manassas, VA, USA).

### Preparation of stock solution of phenolic compounds

The influence of the phenolic compounds was evaluated with different concentrations ranging from 0.5 to 2 mM for coniferyl aldehyde and ferulic acid, and from 0.5 to 10 mM for vanillin and 4-hydroxybenzoic acid. The stock solution was prepared using Milli-Q water (Millipore, Billerica, MA) and the pH was then adjusted to 5.0 with aqueous solutions of sulfuric acid or sodium hydroxide. The concentration of the stock solution was six times higher than the maximum concentration of the phenolic compound in the medium. The stock solutions with coniferyl aldehyde and vanillin were stirred overnight in the dark to achieve complete dissolution. For 4-hydroxybenzoic acid and ferulic acid, a solution of sodium hydroxide was used to adjust the pH of the solution to 5.0, which would help the dissolution of these two compounds. After complete dissolution, an aqueous solution of sulfuric acid was used to adjust the pH to 5.0.

### Cultivation of *G. xylinus*

Liquid seed medium contained 3.0 g/L yeast extract (Merck, Germany), 5.0 g/L tryptone (BD, France), 25 g/L glucose (Fisher Scientific, UK) and Milli-Q water. The pH of all media was adjusted to 5.0. Culture medium was prepared by dissolving 0.75 g glucose, 0.15 g tryptone and 0.09 g yeast extract in 23.2 mL Milli-Q water.

A liquid culture for inoculum was prepared by transferring a bacterial colony grown on agar seed medium (liquid seed medium with 18 g/L agar) into 100 mL of the liquid seed medium, which was then incubated at 30°C with agitation. A cell suspension of 1.8 mL of the inoculum culture was introduced into a 100 mL Erlenmeyer flask containing 23.2 mL of the culture medium. The culture was then incubated at 30°C with agitation for one day. Then, 5 mL of a sterile-filtered (0.2 μm syringe-driven Millex-GN filter unit from Millipore) aqueous stock solution containing the model compound were added to the culture medium. A reference culture without inhibitor was prepared by adding 5 mL autoclaved Milli-Q water instead of the inhibitor solution. A control culture with inhibitor but without bacterial inoculum was prepared as well. The 100-mL Erlenmeyer flasks containing 30 mL of culture medium were then incubated statically at 30°C for 7 days. Samples (2 mL) were withdrawn aseptically from each flask every day during the cultivation.

### Determination of BC yield

Production of BC was quantified gravimetrically based on the dry weight of the insoluble BC harvested at the end of the cultivation. BC was collected by filtration and washed thoroughly with distilled water, and was then dried to constant weight at 105°C. After that, the BC was weighed for calculation of the volumetric yield (g/L) and the yield on consumed sugar (g/g). The yield of BC on consumed sugar (g/g) was calculated using the following equation:

BCyieldonconsumedsugarg/g=BCgGlucoseonfirstdayg−residualglucoseg

### Analysis of glucose consumption

The concentration of glucose during cultivation was monitored by using a glucometer (Accu-Chek Aviva, Roche Diagnostics GmbH, Germany). The consumption rate of the glucose was calculated by using the following equation:

### Determination of phenolic compounds

The concentration of phenols was determined by using a high-performance liquid chromatography (HPLC) instrument (Agilent 1200 series) equipped with a C18 column (Zorbax SB-C18, 3 × 50 mm, 1.8 μm, Agilent Technologies), two pumps (G1312A, Series 1200, Agilent), an autosampler (G1329A, Series 1200, Agilent) and a Diode Array and Multiple Wavelength Detector (G1315D, Series 1200, Agilent). Samples were diluted appropriately with Milli-Q water according to different initial concentrations and were then filtered using 0.2 μm syringe-driven filter units (Millex-GN, Millipore). A total volume of 10 μL of each diluted sample was injected into the C18 column and was circulated for 40 min at a flow rate of 0.4 mL/min. The eluent was a gradient of Milli-Q water and acetonitrile, both of which contained 2 mM formic acid. The concentration of acetonitrile was increased from 0 to 5% within the first 5 min, and further on to 10% after 10 min. After 20 min, the concentration of acetonitrile was 30%, and it was then raised to 50% after 30 min. At the end of the 40 min period, the concentration of acetonitrile was decreased to 5%. The total analysis time was 40 min and the column temperature was maintained at 40°C.

Standard curves for the phenols used in the experiments were prepared within the concentration range 0.5 ppm to 100 ppm. The wavelengths used for quantification were: 254 nm for 4-hydrobenzoic acid and coniferyl alcohol, 280 nm for vanillin, vanillic acid, and vanillyl alcohol, 280 or 330 nm for ferulic acid, and 254 or 330 nm for coniferyl aldehyde.

### Analysis of bacterial viability

The bacteria viability was detected by using a fluorescent dye kit (L7012 Live/Dead BacLight Bacterial Viability Kit from Invitrogen) and a microplate reader (Synergy H4 Hybrid Reader). A standard curve for the relative fluorescence value and the number of bacterial cells was prepared before the determination.

## Competing interests

The authors declare that they have no competing interests.

## Authors’ contributions

All work has been carried out under the supervision of FH and LJJ. SZ carried out most of the experiments, including cultivation of *G. xylinus*, analysis of glucose consumption, pH values, cell viability and BC yield, and drafted the manuscript. SZ, XG and LC investigated phenols added to culture media. SW performed identification and quantification of bioconversion products of added phenols. FH conceived and designed the study and helped to draft the manuscript. LJJ helped to design the study and revised the final manuscript. All authors read and approved the final version of the manuscript.

## Authors’ information

SZ and XG are doctoral candidates with interests in the areas of enzymatic saccharification, microbial cultivation, and bioconversion of biomass for the production of value-added products. SW is a postdoctoral researcher with interests in enzyme chemistry and technology. LC is an associate professor with interests in production and application of bacterial cellulose. FH is a professor with focus on biotechnology and bioengineering for efficient production of bacterial cellulose and enzymes from low-value renewable biomass, as well as applications of BC in biomedicine and functional materials. He was awarded honors of “New Century Excellent Talents in University” by the Ministry of Education of China and “High Level Innovative Talents of Jiangsu” by the Government of Jiangsu Province. LJJ is a professor with focus on biotechnology for biorefining of lignocellulose. He is leader of the Biochemical Platform of the Bio4Energy research initiative (<http://www.bio4energy.se>).
